# Analytic model for the complex effective index of the leaky modes of tube-type anti-resonant hollow core fibers

**DOI:** 10.1038/s41598-017-12234-5

**Published:** 2017-09-18

**Authors:** Matthias Zeisberger, Markus A. Schmidt

**Affiliations:** 1 Leibniz Institute of Photonic Technology, Albert-Einstein-Str. 9, 07745 Jena, Germany; 2 Otto Schott Institute of Materials Research (OSIM), Friedrich Schiller University of Jena, Fraunhoferstr. 6, 07743 Jena, Germany; 3 Abbe Center of Photonics and Faculty of Physics, Friedrich Schiller University Jena, Max-Wien-Platz 1, Jena, 07743 Germany

## Abstract

Due to their promising applications, hollow-core fibers, in particular, their anti-resonant versions, have recently attracted the attention of the photonics community. Here, we introduce a model that approximates, using the reflection of a wave on a single planar film, modal guidance in tube-type anti-resonant waveguides whose core diameters are large compared to the wavelength. The model yields analytic expressions for the real and imaginary parts of the complex effective index of the leaky modes supported, and is valid in all practically relevant situations, excellently matching all the important dispersion and loss parameters. Essential principles such as the fourth power dependence of the modal loss on the core radius at all wavelengths and the geometry-independent transition refractive index, below which modal discrimination favors the fundamental mode are discussed. As application examples, we use our model for understanding higher-order mode suppression in revolver-type fibers and for uncovering the tuning capabilities associated with nonlinear pulse propagation.

## Introduction

Guiding light inside the hollow core of microstructured optical fibers has recently attracted the attention of the photonics community, as such fibers have the potential to open up new fields of science and applications for fiber optics and to achieve new performance levels, examples of which include surgery^1^, mid-IR gas lasers^2,3^, tunable UV light sources^4^, nonlinear optical excitations^5^, high power pulse delivery^6^, photothermal gas trace analysis^7^, remote microparticle sensing^8^, and ultrashort pulse compression^9^. All currently used hollow core fibers (HCFs) have cores with diameters on the order of several tens of micrometers, being larger than those of typical step-index fibers, indicating that upscaling the core diameter allows the current loss limit of telecommunication fibers (e.g., SMF-28: 0.1 dB/km) to be broken^10^. Due to the inverted refractive index (RI) contrast (RI core < RI cladding), modes in HCFs are intrinsically leaky; i.e., they continuously dissipate energy along the transverse direction^11,12^, which is a fundamental difference compared to modes in regular step-index fibers. As a result, HCF research aims to suppress lateral energy dissipation via an as-simple-as-possible microstructure surrounding the core section. Besides guidance mechanisms such as photonic band gap confinement^13^, omnidirectional reflection^14,15^ or effective medium reflection^16,17^, the anti-resonant effect has recently been identified to be highly relevant for future high-performance HCFs, as it enables efficient waveguiding on the basis of a straightforward-to-fabricate microstructure^18,19^. The concept of anti-resonant guidance traces back to close-to-unity reflection of dielectric multilayers in the case of near-grazing incidence and has been successfully employed in, e.g., biosensing^20,21^ or gas analysis^22^ using planar anti-resonant reflecting optical waveguides (ARROWs)^23^. Various types of anti-resonant fiber geometries such as the negative curvature^19,24^, the double revolver^25^, the single ring^26,27^ or the hypocycloid core contour^28^ designs have been recently implemented, exhibiting low optical losses at mid- and near-infrared^28^, visible, and even UV wavelengths^26,27,29^, and low susceptibility to bending^30^. It is common knowledge throughout the fiber optics community that the innermost part of the microstructured section, which in many HCF cases is a ring-type structure, is essential for the overall guidance performance, requiring a precise understanding of leaky mode formation mediated by the central microstructure. For example, ref.^31^ suggests replacing the microstructure of the Kagome design by a simplified design consisting of just a single ring^32,33^. Especially for nonlinear optics, it is vital to obtain precise knowledge of the dispersion of short optical pulses inside the HCFs; e.g., appropriate tuning of the group velocity dispersion allows for multi-octave spanning supercontinuum generation in noble gas filled HCFs^4,34^ or for efficient pulse compression^35,36^. From the attenuation perspective, understanding the dependency of modal attenuation on the core radius *R* is essential, as HCFs allow the loss to be reduced by increasing the core dimensions. This is in great contrast to solid step index fibers, in which material loss dominates, placing a fundamental limit on the loss. For a capillary, it is well known that the loss of the fundamental mode scales with the inverse cube of the core radius ($\propto 1/{R}^{3}$)^16,37^–a behavior that was also observed for Omniguide and indefinite metamaterial HCFs^15,16,38,39^. For a dielectric tube-type waveguide operating at the anti-resonant point, a fourth power dependence of the modal loss on the inverse radius was found in ref.^40^, which was also revealed for a broader spectral region within the microwave and THz domain using an approximate dielectric tube model^41^. Another model that analyzes the modal losses of multilayer waveguide structures was given in ref.^42^. First promising experiments of thin-wall silica capillaries at visible wavelengths clearly indicate the potential of hollow tube-type waveguides with applications in various fields^43^. The results presented in ref.^44^ indicate that the modal attenuation of revolver-type HCFs (which are referred to in the aforementioned paper as tube lattice fibers) scales with 1/*R*^4.5 ^^44^. An approximate model describing anti-resonant fibers has been introduced in ref.^45^ that is applicable for different core shapes but demands numerical integration and does not yield analytic expressions for the complex effective mode index.

**Figure 1:**
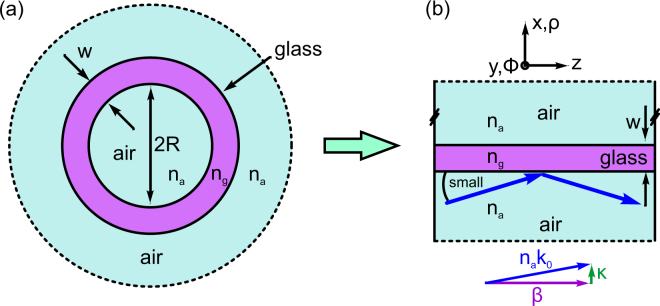
(**a**) Cross section of the tube-type anti-resonant hollow core fiber geometry discussed here, consisting of an air core, a glass ring and an air cladding with all relevant parameters (light blue: air, purple: glass. *n*_*a*_ and *n*_*g*_: refractive indices of air and glass). (**b**) Planar single layer reflection model, including a single wave (indicated by the blue arrows) reflected on a planar glass film from the interior air side. The various arrows below the sketch represent the wave vector diagram of the propagating mode, i.e., of the wave incident on the glass film (purple: propagation constant, blue: wave vector of incident wave, green: transverse wave vector).

Here we introduce a planar reflection model that approximates tube-type anti-resonant HCFs by single planar films (Fig. 1), yielding entirely analytic expressions for the complex effective index of the leaky modes in such HCFs whose core radius is much larger than the wavelength. We compare our model to numerically obtained solutions, showing that it describes the real *and* imaginary parts of the complex effective index extremely well across the entire bandwidth of the transmission bands down to core diameters that are below those of practically relevant anti-resonant HCFs. Excellent agreement was found for the most important dispersion parameters (i.e., the phase velocity, group velocity, and group velocity dispersion) and for the imaginary parts of the effective indices. The model reveals the essential features of light guidance in tube-type waveguides, examples of which include a fourth power dependence of the modal loss on core radius and a transition glass index above which modal discrimination counterintuitively favors the TE_01_ mode, even though the HE_11_ mode has the greatest phase index. Here, we apply our model to two important topics of HCF research, namely, to the suppression of higher-order modes in revolver-type fibers via phase-matching them to cladding modes and to zero-dispersion wavelength (ZDW) tuning capabilities of gas-filled tube-type HCFs.

## Results

The general principle for light guidance in a tube-type anti-resonant HCF relies on the reflection of a single wave at the inner and outer surfaces of the glass ring and the constructive interference of both reflected waves. It is important to note that close-to-unity reflection values ($|r{|}^{2}\lesssim 1$, *r*: amplitude reflection coefficient) are obtained in the case of near-grazing incidence even for such a comparably simple geometry, which forms one of the underlying mechanisms for the low loss of anti-resonant HCFs. Comparable to our approach presented in ref.^16^ we use this particular property and expand the amplitude reflection coefficient of a single planar interface with respect to the incidence angle, assuming near-grazing incidence. The resulting expression for the reflection coefficient is then included in approximate formulations of the field distributions of the leaky core modes, yielding an entirely analytical expression for the complex effective index of the leaky modes of this geometry.

### Fields in the core and zero-order approximation

The fields in the core are determined by the solutions of the Helmholtz equation in cylindrical coordinates^46^. Modes are formed by taking into account the continuity of the transverse field components across the layer boundaries. Due to the azimuthal invariance of the cylindrical geometry, it is sufficient to analyze the radial distributions of the longitudinal and azimuthal components of the electric and magnetic fields (*z* and $\phi$ directions), given by

1 $${E}_{z}(\rho )={A}_{1}{J}_{m}(\kappa \rho ),\,{H}_{z}(\rho )={A}_{2}{J}_{m}(\kappa \rho ),$$

2 $${E}_{\phi }(\rho )=-\frac{\beta m}{{\kappa }^{2}\rho }{A}_{1}{J}_{m}(\kappa \rho )-\frac{i{Z}_{0}{k}_{0}}{\kappa }{A}_{2}{J}_{m}^{^{\prime} }(\kappa \rho \mathrm{),\ \ }{H}_{\phi }(\rho )=\frac{i{n}_{a}^{2}{k}_{0}}{{Z}_{0}\kappa }{A}_{1}{J}_{m}^{^{\prime} }(\kappa \rho )-\frac{\beta m}{{\kappa }^{2}\rho }{A}_{2}{J}_{m}(\kappa \rho ),$$

3 $${\beta }^{2}={k}_{0}^{2}{n}_{eff}^{2}={k}_{0}^{2}{n}_{a}^{2}-{\kappa }^{2},$$

with the axial and radial wave vector components being *β* and $\kappa$, the vacuum wave number *k*_0_, the core and outermost RI *n*_*a*_, the radial coordinate $\rho$, the azimuthal mode index *m*, the vacuum impedance *Z*_0_, and the amplitudes *A*_1_ and *A*_2_ (full expressions for the fields are given in the Methods section). As shown in our previous work^16^, the effect of the media or microstructure surrounding the core is subsumed in the polarization-dependent amplitude reflection coefficients *r*_*e*_ and *r*_*m*_ (*e*: TE pol., *m*: TM pol.). Using these quantities, the following conditions for the fields at the core boundary ($\rho =R$) are derived (see^16^ for details):

4 $$\frac{{E}_{\phi }}{{H}_{z}}=\frac{{Z}_{0}{k}_{0}}{\kappa }\frac{1+{r}_{e}}{1-{r}_{e}},\,\frac{{H}_{\phi }}{{E}_{z}}=\frac{{k}_{0}{n}_{a}^{2}}{{Z}_{0}\kappa }\frac{{r}_{m}+1}{{r}_{m}-1}$$

Low fiber losses imply a high layer reflectivity, i.e., a reflection coefficient ${r}_{e/m}\approx -1$ (the reflection coeffi-cient is defined here as the ratio of the reflected (r) and incident (i) transverse field components: ${r}_{e}={E}_{\phi }^{(r)}/{E}_{\phi }^{(i)},{r}_{m}={H}_{\phi }^{(r)}/{H}_{\phi }^{(i)}$). In the limiting case of a perfectly reflecting interface (${r}_{e}={r}_{m}=-1$) Eq. (4) reduce to ${E}_{\phi }=0$ and ${H}_{\phi }=0$ resulting in the following solutions for the radial wavenumber^16^:

5 $$\kappa =\{\begin{array}{ll}{j}_{1n}/R & {\rm{for}}\,{{\rm{TE}}}_{0n}\,{\rm{or}}\,{{\rm{TM}}}_{0n}\,{\rm{modes}}\\ {j}_{m-\mathrm{1,}n}/R & {\rm{for}}\,{{\rm{HE}}}_{mn}\,{\rm{modes}}\\ {j}_{m+\mathrm{1,}n}/R & {\rm{for}}\,{{\rm{EH}}}_{mn}\,{\rm{modes}}\end{array}$$

where *j*_*mn*_ is the *n*-th root of the Bessel function *j*_*m*_. In the general case of non-unity reflectivity ($|r|\lesssim 1$), the solution for the radial wave number can be obtained by inserting Eqs. (1, 2) into Eq. (4) which results in a homogeneous system of equations for the amplitudes *A*_1_ and *A*_2_:

6 $$M(\begin{array}{l}{A}_{1}\\ {A}_{2}\end{array})=(\begin{array}{l}0\\ 0\end{array})$$

with the coefficient matrix

7 $$M=(\begin{array}{ll}m({r}_{e}-\mathrm{1)}\beta {J}_{m}(\kappa R) & {k}_{0}R\kappa [\mathrm{(1}+{r}_{e}){J}_{m}(\kappa R)+i\mathrm{(1}-{r}_{e}){J}_{m}^{^{\prime} }(\kappa R)]\\ {k}_{0}{n}_{a}^{2}R\kappa [\mathrm{(1}+{r}_{m}){J}_{m}(\kappa R)+i\mathrm{(1}-{r}_{m}){J}_{m}^{^{\prime} }(\kappa R)] & m\beta ({r}_{m}-\mathrm{1)}{J}_{m}(\kappa R)\end{array})\mathrm{.}$$

The values of $\kappa$ that correspond to the modes of the cylindrical fiber waveguide can be obtained by finding the nontrivial solutions of Eq. (6), imposing the condition det(*M*) = 0.

### Reflection at a planar film

In order to obtain the reflection coefficients we consider a small volume element of size ${\rm{\Delta }}\rho \times \rho {\rm{\Delta }}\phi \times {\rm{\Delta }}z$ at the boundary of the core that includes the inner and the outer surfaces of the glass ring (Fig. 1b). If ${\rm{\Delta }}\rho \ll R$ and ${\rm{\Delta }}\phi \ll 1$, we can neglect the curvature of the surfaces of the glass ring, allowing us to approximate the considered volume as a symmetric planar film and the fields as a superposition of the incident and reflected plane waves. The reflection coefficients of the TE/TM waves for such a film are given by the Airy formula, which fundamentally describes a Fabry–Perot-type structure^47^:

8 $${r}_{e/m}=\frac{{\tilde{r}}_{e/m}(1-\exp \mathrm{(2}i\phi ))}{1-{\tilde{r}}_{e/m}^{2}\exp \mathrm{(2}i\phi )}\mathrm{.}$$

The relative phase $\phi$ corresponds to the accumulated phase acquired between two reflection events of a single wave inside the film of thickness $w$ and is given by

9 $$\phi ={\kappa }_{g}w,\quad {\kappa }_{g}=\sqrt{{k}_{0}^{2}({n}_{g}^{2}-{n}_{a}^{2})+{\kappa }^{2}},$$

with $\kappa$ and ${\kappa }_{g}$ being the wave vector components perpendicular to the film surface in air and glass, respectively. The parameters ${\tilde{r}}_{em}$ are the polarization-dependent amplitude reflection coefficients of a single air–glass interface and are given by the Fresnel equations^47^:

10 $${\tilde{r}}_{e/m}=\frac{{\eta }_{e/m}\kappa /{\kappa }_{g}-1}{{\eta }_{e/m}\kappa /{\kappa }_{g}+1},\quad {\eta }_{e}=\mathrm{1,}\quad {\eta }_{m}=\varepsilon ,\quad \varepsilon =\frac{{n}_{g}^{2}}{{n}_{a}^{2}}\mathrm{.}$$

It is evident from Eq. (8) that the main spectral characteristics of a thin film, i.e., its resonant behavior, result from the relative phase $\phi$, especially in the case of low material dispersion. For $\phi ={\phi }_{l}^{(r)}=l\pi$ with $l=\mathrm{1,}\,\mathrm{2,}\,\mathrm{...}$, the optical wave is in resonance with the film; i.e., the reflection coefficient is zero, which in the case of a fiber waveguide corresponds to an infinitely high loss. The maximum reflectivity (i.e., the lowest possible fiber attenuation) occurs for the anti-resonant condition $\phi ={\phi }_{l}^{(a)}=\mathrm{(2}l-\mathrm{1)}\pi \mathrm{/2}$. The main approximation of our model is that the core diameter is much larger than the operational wavelength ($R\gg \lambda$), leading to a radial wavenumber in the core that is small compared to the wavenumber of the wave considered ($\kappa \ll {k}_{a}={k}_{0}{n}_{a}$) and yielding the near-grazing incidence condition. For this situation we can expand the amplitude reflection coefficients of the single interface ${r}_{e/m}$ with respect to $\kappa$ up to the second-order term and obtain the following expression:

11 $${r}_{e/m}=-1+\frac{2i{\eta }_{e/m}\,\cot \,\phi }{{k}_{a}\sqrt{\varepsilon -1}}\kappa +\frac{\mathrm{(2}+4{\cot }^{2}\phi ){\eta }_{e/m}^{2}}{{k}_{a}^{2}(\varepsilon -\mathrm{1)}}{\kappa }^{2}+O({\kappa }^{3})\mathrm{.}$$

In addition, we obtain the following approximation for the phase

12 $$\phi ={k}_{0}w\sqrt{{n}_{g}^{2}-{n}_{a}^{2}}(1+O{(\frac{\kappa }{{k}_{0}})}^{2})\mathrm{.}$$

In the case of a glass capillary (i.e., no outer glass–air boundary; corresponding to a single planar air–glass interface), we have shown in a previous contribution that the linear approximation of ${r}_{e/m}$ is sufficient to calculate the modal losses^16^, yielding the well-known Marcatili expression^37^. In the case of the tube-type anti-resonant fiber, however, Eq. (11) shows that the linear term is entirely imaginary, indicating that this term merely causes a phase shift and has no influence on the modal losses. It is important to note that at the anti-resonance point (${\phi }_{l}^{(a)}$) the linear term completely vanishes (as $\cot (\phi )=0$), and the linear approximation of ${r}_{e/m}$ is not sufficient to calculate the losses of an anti-resonant fiber, requiring inclusion of the next higher-order expansion term that is proportional to ${\kappa }^{2}$.

### Perturbation treatment

In the following, we derive an approximate solution for the complex effective index ${n}_{eff}$ of the lowest-order modes in the case of high layer reflectivity ($|{r}_{e/m}|\lesssim -1$). The perturbation parameter we use here is $\sigma =1/({k}_{a}R)\propto \lambda /R\ll 1$ (with the core wavenumber ${k}_{a}$) which is small for the condition considered here ($R\gg \lambda$). We use the following ansatz for the radial wavenumber:

13 $$\kappa ={k}_{a}[{\kappa }_{1}\sigma +{\kappa }_{2}{\sigma }^{2}+{\kappa }_{3}{\sigma }^{3}+O({\sigma }^{4})],$$

which is consistent with the solution for the perfectly reflecting interface (Eq. (5)). To obtain solutions for ${n}_{eff}$ Eq. (13) is inserted into Eq. (11) and the result into the system of equations (Eq. (6)). After a third-order series expansion of the resulting equations with respect to $\sigma$, the coefficients ${\kappa }_{1}$, ${\kappa }_{2}$ and ${\kappa }_{3}$ can be obtained by equating the coefficients of $\sigma$. The series expansions result in derivatives of Bessel functions up to the third order which are reformulated using the equations shown in the Methods section. Using the solution for the radial wavenumber the effective index can be calculated by the following expression:

14 $${n}_{eff}={n}_{a}\sqrt{1-\frac{{\kappa }^{2}}{{k}_{a}^{2}}}={n}_{a}[1-\frac{{\kappa }_{1}^{2}}{2}{\sigma }^{2}-{\kappa }_{1}{\kappa }_{2}{\sigma }^{3}-(\frac{{\kappa }_{1}^{4}}{8}+\frac{{\kappa }_{2}^{2}}{2}+{\kappa }_{1}{\kappa }_{3}){\sigma }^{4}]+O({\sigma }^{5})\mathrm{.}$$

Detailed calculations for the different mode types show that ${\kappa }_{1}$ and ${\kappa }_{2}$ are real-valued, whereas ${\kappa }_{3}$ is complex (in the case of real material parameters). As a consequence, we are allowed to express ${n}_{eff}$ using four real parameters (labeled as *a*, *b*, *c*, and *d*):

15 $${n}_{eff}={n}_{a}[1-a{\sigma }^{2}-b{\sigma }^{3}-c{\sigma }^{4}+id{\sigma }^{4}]+O({\sigma }^{5}),$$

16 $$a=\frac{{\kappa }_{1}^{2}}{2},\quad b={\kappa }_{1}{\kappa }_{2},\quad c=\frac{{\kappa }_{1}^{4}}{8}+\frac{{\kappa }_{2}^{2}}{2}+{\kappa }_{1}{\rm{Re}}\,{\kappa }_{3},\quad d=-{\kappa }_{1}{\rm{Im}}\,{\kappa }_{3}\mathrm{.}$$

### TE and TM modes

The azimuthal invariance of the cylindrical polarized modes leads to a vanishing azimuthal mode index (*m* = 0)^48^, considerably simplifying the derivation of the effective index. In the case of TE_0*n*_ modes, *A*_1_ = 0, which reduces the system of equations (Eq. (6)) to the single equation:

17 $$\mathrm{(1}+{r}_{e}){J}_{0}(\kappa R)+i\mathrm{(1}-{r}_{e}){J}_{0}^{^{\prime} }(\kappa R)=0.$$

A similar equation is obtained for the TM_0*n*_ modes (*A*_2_ = 0) by substituting *r*_*e*_ by *r*_*m*_. The calculation for both types of modes results in the following expressions for the expansion coefficients

18 $${\kappa }_{1}={j}_{1n},\quad {\kappa }_{2}=\frac{{\eta }_{e/m}{j}_{1n}\,\cot \,\phi }{\sqrt{\varepsilon -1}},\quad {\kappa }_{3}=\frac{{j}_{1n}^{2}{\eta }_{e/m}^{2}}{\varepsilon -1}[\frac{3}{2}\,{\cot }^{2}\phi -i\mathrm{(1}+{\cot }^{2}\phi )],$$

from which we obtain the coefficients for Eq. (15):

19 $$b={b}_{0}\frac{\cot \,\phi }{\sqrt{\varepsilon -1}}\cdot \{\begin{array}{ll}1 & {\rm{TE}}\\ \varepsilon  & {\rm{TM}}\end{array},\quad c={c}_{0}+{c}_{1}\frac{{\cot }^{2}\phi }{\varepsilon -1}\cdot \{\begin{array}{ll}1 & {\rm{TE}}\\ {\varepsilon }^{2} & {\rm{TM}}\end{array},\quad d={d}_{0}\frac{1+{\cot }^{2}\phi }{\varepsilon -1}\cdot \{\begin{array}{ll}1 & {\rm{TE}}\\ {\varepsilon }^{2} & {\rm{TM}}\end{array}$$

with the constants

20 $$a=\frac{{j}_{1n}^{2}}{2},\quad {b}_{0}={j}_{1n}^{2},\quad {c}_{0}=\frac{{j}_{1n}^{4}}{8},\quad {c}_{1}=2{j}_{1n}^{2}\mathrm{,\ \ }{d}_{0}={j}_{1n}^{3}\mathrm{.}$$

### Hybrid modes

In the case of HE_*mn*_ and EH_*mn*_ modes, the values of $\kappa$ are obtained by solving det(*M*) = 0. With the perturbation ansatz (Eq. (13)) we obtain the following expressions for the expansion coefficients of the radial wavenumber:

21 $${\kappa }_{1}={j}_{m+s,n},\quad {\kappa }_{2}=\frac{{j}_{m+s,n}}{2\sqrt{\varepsilon -1}}({\eta }_{e}+{\eta }_{m})\cot \,\phi ,$$

22 $${\kappa }_{3}=\frac{{j}_{m+s,n}ms}{2}+[{j}_{m+s,n}(\frac{3}{8}+\frac{ms}{4}){({\eta }_{e}+{\eta }_{m})}^{2}-\frac{{j}_{m+s,n}^{3}s}{8m}{({\eta }_{e}-{\eta }_{m})}^{2}]\frac{{\cot }^{2}\phi }{\varepsilon -1},$$

where $s=-1$ refers to HE and $s=+1$ to EH modes. The effective mode index is represented by the same expression as for the TE/TM modes (Eq. (15)) with the following parameters and constants:

23 $$b={b}_{0}\frac{\varepsilon +1}{\sqrt{\varepsilon -1}}\,\cot \,\phi ,\,c={c}_{0}+[{c}_{1}\frac{{(\varepsilon +\mathrm{1)}}^{2}}{\varepsilon -1}-{c}_{2}(\varepsilon -\mathrm{1)}]\,{\cot }^{2}\phi ,\,d={d}_{0}\frac{{\varepsilon }^{2}+1}{\varepsilon -1}(1+{\cot }^{2}\phi ),$$

24 $$a=\frac{{j}_{m+s,n}^{2}}{2},\,{b}_{0}=\frac{{j}_{m+s,n}^{2}}{2},\,{c}_{0}=\frac{{j}_{m+s,n}^{4}}{8}+\frac{{j}_{m+s,n}^{2}ms}{2},\,{c}_{1}=\frac{{j}_{m+s,n}^{2}}{4}\mathrm{(2}+ms),\,{c}_{2}=\frac{{j}_{m+s,n}^{4}}{8m},\,{d}_{0}=\frac{{j}_{m+s,n}^{3}}{2}\mathrm{.}$$

## Discussion

The calculations in the previous sections show that Eq. (15) describes the complex effective index of all modes, with the coefficients *a*, *b*, *c*, and *d* being real numbers that depend on the type and indices (*m*, *n*) of the mode under investigation. The second-order approximation given by the terms $1-a{\sigma }^{2}$ represents the limiting case of a lossless cylindrical fiber, i.e., a tube waveguide with a perfectly reflecting surface. The third-order term $b{\sigma }^{3}$ in Eq. (15) describes resonant effects in the real part of *n*_*eff*_. The $c{\sigma }^{4}$ term is an additional contribution to the real part, which is in all cases considered here small compared to $b{\sigma }^{3}$ and can be neglected for all practical purposes. The last term in Eq. (15) represents the imaginary part of the effective index (Im $({n}_{eff})={n}_{a}d{\sigma }^{4}$) which is proportional to the modal loss and scales here with $\mathrm{1/}{R}^{4}$, which is remarkably different from other types of HCF such as photonic band gap fibers. For the case of operation at the anti-resonant point ($\cot \,\phi =0$), the imaginary part corresponds to the results shown in ref.^40^, whereas an excellent match to the loss expressions reported in ref.^41^ was also found. It is important to note that the loss of tube lattice fibers scales with *R*^−4.5^ (as numerically shown in ref.^44^), which is very close to the radius dependence observed here. By inserting the correct terms into Eq. (15), we obtain the following analytic expressions for the real part of the effective index (i.e., the phase index Re (*n*_*eff*_)) and the loss coefficient *α* (given in m^−1^):

25 $${\rm{Re}}({n}_{eff})={n}_{a}-\frac{{j}^{2}}{2{k}_{0}^{2}{n}_{a}{R}^{2}}-\frac{{j}^{2}}{{k}_{0}^{3}{n}_{a}^{2}{R}^{3}}\frac{\cot \,\phi }{\sqrt{\varepsilon -1}}\cdot \{\begin{array}{ll}1 & {{\rm{TE}}}_{0n}\\ \varepsilon  & {{\rm{TM}}}_{0n}\\ (\varepsilon +\mathrm{1)/2} & {\rm{HE}}/{{\rm{EH}}}_{mn}\end{array}$$

26 $$\alpha =\frac{1+{\cot }^{2}\phi }{\varepsilon -1}\frac{{j}^{3}}{{k}_{0}^{3}{n}_{a}^{3}{R}^{4}}\cdot \{\begin{array}{ll}1 & {{\rm{TE}}}_{0n}\\ {\varepsilon }^{2} & {{\rm{TM}}}_{0n}\\ ({\varepsilon }^{2}+\mathrm{1)}/2 & {\rm{HE}}/{{\rm{EH}}}_{mn}\end{array}{\rm{with}}\quad j=\{\begin{array}{ll}{j}_{1n} & {\rm{TE}}/{{\rm{TM}}}_{0n}\\ {j}_{m-\mathrm{1,}n} & {{\rm{HE}}}_{mn}\\ {j}_{m+\mathrm{1,}n} & {{\rm{EH}}}_{mn}\end{array}$$

It is important to note that our work focusses on tube-type waveguides in which the losses arise only from leaky waves rather than material absorption, thus allowing to understand the influence of non-unity interface reflection on modal attenuation. As a consequence the coefficients *a*, *b*, *c*, and *d* in Eq. (15) are real valued for real RIs which allows to clearly distinguish the real and imaginary parts in Eq. (15). However, the derivation presented in the previous section is not restricted to real valued *n*_*a*_ and *n*_*g*_ and Eq. (15) is valid for materials with non-negligible imaginary parts as well. In this case, the coefficients *a*, *b*, *c*, and *d* become complex, and the clear separation of the real and imaginary terms in Eq. (15) is not obvious anymore. We have checked various numerical examples including complex material permittivities using Eqs (15, 19, 23) and found a very good agreement of Im(*n*_*eff*_) with results obtained from the equations given in ref.^42^. The final equations for Re(*n*_*eff*_) and $\alpha$ (Eqs (25) and (26)) are derived from the previous equations assuming real RIs and thus are valid for non-absorbing materials only.

**Figure 2:**
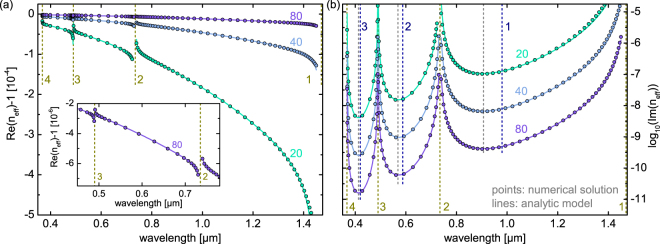
Comparison of the spectral distributions of the real (**a**) and imaginary (**b**) parts of the effective mode index of the fundamental leaky HE_11_ mode supported by the tube-type anti-resonant hollow core fiber of model (lines) and full numerical solution (circles) for different core radii (light green: $R=20\,\mu {\rm{m}}$, blue: $R=40\,\mu {\rm{m}}$, purple: $R=80\,\mu {\rm{m}}$). The simulated structure assumes thickness and refractive index values of the tube of $w=0.7\,\mu {\rm{m}}$ and ${n}_{g}=1.45$ and a refractive index of unity in the core and outermost medium. The inset in (**a**) is a close-up view of the distribution with $R=80\,\mu {\rm{m}}$ in the vicinity of the two resonances. In all plots, the dark yellow vertical dashed lines indicate the resonances, the gray lines the wavelength of minimal loss and the dark blue lines the anti-resonant wavelength. The dark blue (dark yellow) numbers indicate the respective anti-resonance (resonance) order.

To verify the validity of our planar reflection model, we compared spectral distributions of the complex RI dispersion of the fundamental leaky mode (HE_11_ mode) of a single silica tube in air calculated using Eq. (15) (solid lines in Fig. 2) with full numerical solutions (points in Fig. 2) for three different core radii. Both real (Fig. 2a) and imaginary (Fig. 2b) parts of the numerical solutions are fully reproduced by our analytical model (Eqs (15, 23–24)) for all bands considered, even close to the resonances, indicating that the expansion of $\kappa$ up to the third-order term is sufficient to describe the modal behavior of tube-type anti-resonant HCFs. In particular, the extremely good match in the imaginary parts is remarkable (Fig. 2b). Only in very close proximity to the resonances (corresponding to practically irrelevant domains due to the exceedingly high loss), particularly on the long-wavelength side of the lowest-order transmission band, does the planar model slightly deviate from the numerical solutions, which overall is due to the reduced reflectivity of the thin glass layer, i.e., from the breakdown of the condition of high reflectivity ($|{r}_{e/m}+\mathrm{1|}\ll 1$). From the practical perspective, HCFs are always operated far away from the resonances in order to avoid excessive modal loss.

**Figure 3:**
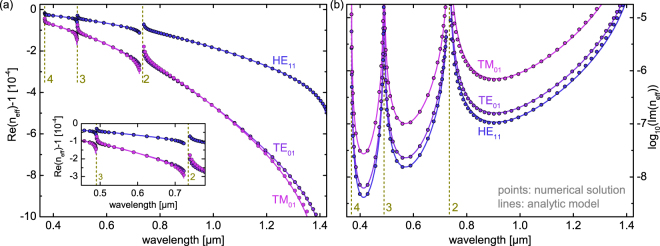
Comparison of the spectral distributions of the real (**a**) and imaginary (**b**) parts of the effective mode index of the three lowest-order modes supported by the tube-type anti-resonant hollow core fiber (blue: HE_11_, purple: TE_01_, pink: TM_01_, $R\,=\,20\,\,\mu {\rm{m}}$, $w\,=\,0.7\,\mu {\rm{m}}$, *n*_*g*_ = 1.45, *n*_*a*_ = 1, lines: model, circles: numerical solution). In both plots, the dark yellow vertical dashed lines indicate the resonances with the corresponding order. The inset in (**a**) is a close-up view of the spectral interval between the second and third-order resonances.

It is interesting to note that the spectral positions of minimum loss (gray dashed lines in Fig. 2b) and of the corresponding anti-resonance wavelengths (dark blue dashed lines in Fig. 2b) generally do not coincide, which is particularly pronounced for the longest-wavelength transmission band. As an example in the case of $l=1$, the anti-resonance wavelength is 980 nm, whereas the wavelength of minimal loss is located about 70 nm towards the shorter wavelengths (908 nm). This effect emerges as a consequence of Eqs (15, 23): The positions of the anti-resonance points are given by the minima of the coefficient *d* which depends on $\lambda$ via ${\cot }^{2}\phi$. The factor ${\sigma }^{4}$ of the imaginary part of *n*_*eff*_ contains an additional factor ${\lambda }^{4}$ that shifts the loss minima towards shorter wavelengths. As the next step, we analyzed the spectral distribution of the complex effective index for the three lowest-order modes (HE_11_: blue, TE_01_: purple, TM_01_: magenta) at a fixed core radius of $R=20\,\mu {\rm{m}}$ (Fig. 3a,b). Again, excellent agreement between the planar reflection model and numerical solutions across almost the entire bandwidth of the individual transmission band is obtained, confirming once more the applicability of the planar reflection model for describing guidance in tube-type anti-resonant HCFs.

**Figure 4:**
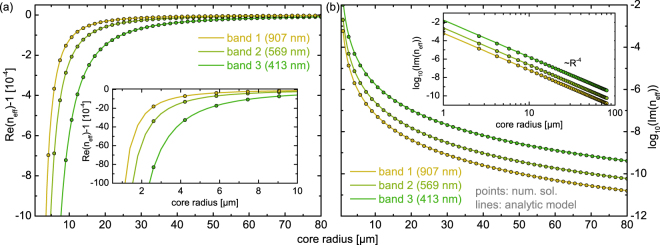
Comparison of the radius dependence of the real (**a**) and imaginary (**b**) parts of the effective mode index of the fundamental leaky HE_11_ mode supported by the tube-type anti-resonant hollow core fiber at the wavelengths of lowest loss of the three investigated transmission bands (lines: model, circles: numerical solution, light yellow: band 1, light green: band 2, green: band 3). The corresponding wavelengths are indicated in the plots. The inset in (**a**) shows the radius dependence in a reduced radius interval between 1 *μ*m and 10 *μ*m. The inset shown in (**b**) is a double-logarithmic representation of the radius dependence of Im (*n*_*eff*_) at the three wavelengths considered, emphasizing the *R*^−4^ dependence.

It is particularly important to examine the wavelengths of lowest loss, which are roughly located within the center of the respective transmission band and are independent of the core diameter as revealed by our analysis. Starting from large core diameters ($R=80\,\mu {\rm{m}}$), the real part of the complex effective index substantially reduces towards smaller cores (Fig. 4a), whereas Im (*n*_*eff*_) increases by orders of magnitude (Fig. 4b), with both effects being fully reproduced by our model for any of the core diameters considered. It is remarkable that the agreement holds even for core diameters $< 5\,\mu {\rm{m}}$ - a diameter range being below that of any practically relevant HCF - emphasizing that guidance in tube-type anti-resonant HCFs is well described by a reflection process of a wave on a single planar interface. As a consequence of this agreement, the imaginary part of Eq. (15) directly reveals that the loss of tube-type anti-resonant HCFs scales with *R*^−4^ (inset of Fig. 4b), which is to some extent unexpected due to the inverse cube dependence observed in other types of HCFs, but is a direct consequence of the vanishing linear term in the expansion of the film reflection coefficient ${r}_{e/m}$ (Eq. (11)) in the case of anti-resonance, requiring inclusion of the quadratic term. It should be mentioned that, compared to the tube-type geometry investigated here, revolver-type anti-resonant fibers^19,24^ can show significantly lower losses which can be qualitatively understood in context of our model as a higher interface reflection caused by the negative curvature of the core region. As we investigate here light guidance in tube-type waveguides from a general perspective, we omit a direct comparison to concrete single-ring hollow core fiber designs which would be beyond the scope of this work.

**Figure 5:**
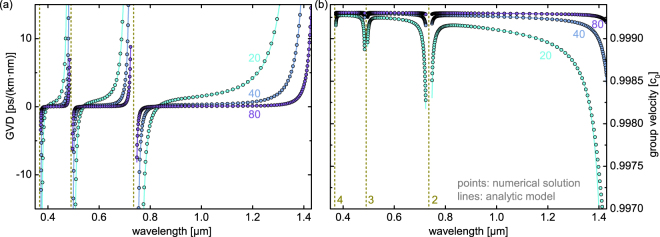
Comparison of the spectral distributions of (**a**) group velocity dispersion and (**b**) group velocity of the fundamental leaky mode of the tube-type anti-resonant fiber for three different core radii (light green: 20 *μ*m, blue: 40 *μ*m, purple: 80 *μ*m; lines: reflection model; circles: full numerical solution; $w=0.7\,\mu {\rm{m}}$; ${n}_{g}=1.45$; RIs of core and cladding are unity; material dispersions have been neglected here). In both plots, the dark yellow vertical dashed lines represent the resonances.

Especially from the ultrafast nonlinear optics perspective, precise knowledge on pulse dispersion is required to optimize the nonlinear conversion processes. For instance, tuning group velocity dispersion (GVD) in a tailored way has allowed for multi-octave spanning supercontinuum generation down to UV wavelengths using ultrafast light-matter interaction in noble gas-filled Kagome or anti-resonant HCFs^4,34^. Moreover, accurate knowledge of the spectral evolution of the group velocity (GV) is important for parametric processes in case femtosecond pulses are employed^49^. Here we determine the spectral distribution of both the GVD and the GV of the tube-type leaky waveguide geometry (Fig. 5a,b) by using the real part of the effective index of the fundamental leaky mode (GVD $=-{\omega }_{0}^{2}/\mathrm{(2}\pi {c}_{0})\cdot ({d}^{2}\beta /d{\omega }^{2}{)|}_{\omega ={\omega }_{0}}$; ${v}_{g}={((d\beta /d\omega {)|}_{\omega ={\omega }_{0}})}^{-1})$ for the three different core radii used in Fig. 2. An extremely good match of both GVD and GV calculated using the planar reflection model and full numerical simulations has been obtained across all three transmission bands for all the core radii considered. It is important to highlight the excellent match of the GVD, as this parameter does not depend on the absolute value of Re (*n*_*eff*_) but rather on the shape of the dispersion curve. As a result of the excellent match in both the GVD and the GV, it is apparent that our reflection model and the derived analytic expression of the complex effective index (Eqs (25, 26)) correctly describe the dispersion properties of the tube-type anti-resonant fibers. The mathematical form of Eq. (25) intuitively suggests that away from the resonances, the dispersion of tube-type cylindrical fibers corresponds to a tube waveguide with a perfectly reflecting cladding (first two terms in Eq. (25)), which is modified by an additional resonance term (the third term in Eq. (25)) within the spectral vicinities of the resonances.

Many applications of HCFs require strong fundamental mode discrimination such that the fundamental leaky mode with a Gaussian-type field profile inside the core has the lowest loss of all modes supported with as high as possible loss discrimination for all other higher-order modes (HOMs)^24,26^. Efficient modal discrimination is critically important in the case of HCFs, as the number of modes in leaky waveguides cannot be controlled in the same a way as for waveguides supporting guided modes^11^. Our planar reflection model allows formulation of analytic expressions for the modal discrimination of the fundamental mode with respect to the HOMs using the various imaginary parts of Eq. (15), which in the case of the three lowest-order modes (HE_11_, TE_01_ and TM_01_), leads to

27 $${c}_{TE}=\frac{{n}_{eff,TE01}^{^{\prime\prime} }}{{n}_{eff,HE11}^{^{\prime\prime} }}=\frac{2{j}_{11}^{3}}{{j}_{01}^{3}({\varepsilon }^{2}+\mathrm{1)}},\,{c}_{TM}=\frac{{n}_{eff,TM01}^{^{\prime\prime} }}{{n}_{eff,HE11}^{^{\prime\prime} }}=\frac{2{j}_{11}^{3}{\varepsilon }^{2}}{{j}_{01}^{3}({\varepsilon }^{2}+\mathrm{1)}}\mathrm{.}$$

**Figure 6:**
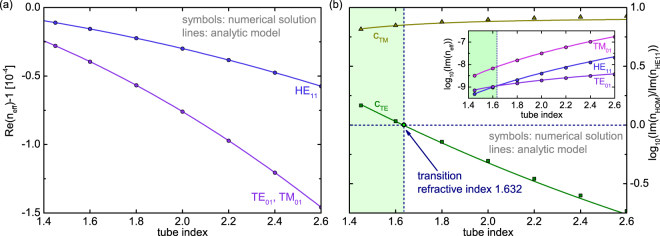
(**a**) Dispersion of the three lowest-order leaky modes as a function of the tube refractive index (blue: HE_11_, purple: TE_01_, magenta: TM_01_). Because the dispersions of the TE_01_ and TM_01_ modes fully overlap, the TM_01_ curve is not visible. (**b**) Modal discrimination of the TE_01_ and TM_01_ modes with respect to the HE_11_ mode (green: TE-discrimination, dark yellow: TM-discrimination) as a function of tube index. The purpose of the dashed blue lines is to guide the eye; they highlight the transition refractive index (indicated by the green dot) and separate the regions of the HE_11_ (light green) and TE_01_ (white) mode, being the lowest loss modes in the system. The inset shows the evolution of Im $({n}_{eff})$ as a function of the tube index for the HE_11_ (blue), TE_01_ (purple), and TM_01_ (magenta) modes. The results in all plots have been calculated at the anti-resonance point of the longest wavelength transmission band assuming $R=80\,\mu {\rm{m}}$ and ${n}_{a}=1$ (symbols: numerical solutions; lines: planar reflection model).

Such relations were also discussed in refs^40,41^. for the special case of operation at the anti-resonant point (i.e., $\cot \,\phi =0$), whereas we show here that these relations are valid at all wavelengths. The discrimination parameters *c*_*TE*_ and *c*_*TM*_ only include constants and the material dielectric functions and are independent of fiber geometry and wavelength (in the case of negligible material dispersion). In particular, they do not depend on the core radius which to some extent is counterintuitive. We analyzed the modal discrimination by calculating Im (*n*_*eff*_) as function of the RI of the tube for the three modes considered using numerical solutions and our reflection model (inset of Fig. 6b), obtaining an excellent agreement and thereby justifying the validity of Eq. (27). Analyzing the modal discriminations as functions of *n*_*g*_ reveals an interesting feature (Fig. 6b), which was also observed for the specific situation of the anti-resonant point in refs^40,41^: within the RI interval between 1.45 and 1.632 (the green area in Fig. 6), the HE_11_ mode has the lowest loss of all modes in the system. For larger RIs, however, the loss of the HE_11_ mode exceeds that of the TE_01_ mode and remains above it for any higher value of *n*_*g*_ (the white area in Fig. 6). This effect, which was confirmed by numerical simulations (symbols in Fig. 6), is not visible in the dispersions of the modes (i.e., the real parts of ${n}_{eff}$), showing that the HE_11_ mode has the highest effective index for any value of ${n}_{g}$ (Fig. 6a). Overall, this unusual discrimination behavior underscores an important conclusion: RIs below the transition value of 1.632 are favorable for tube-type anti-resonant HCFs, as higher RIs impose insufficient modal discrimination of the HE_11_ mode on HOMs. A similar type of discrimination behavior at a transition RI of 2.02 was found for capillary-type waveguides^37^. Compared to the tube-type waveguide geometry discussed here, the larger value of the transition index can mathematically be explained by the different powers of the zeros of Bessel function in the expressions for the attenuation (${j}^{3}$ for tube-type waveguide (Eq. (26)), ${j}^{2}$ for capillaries^37^).

**Figure 7:**
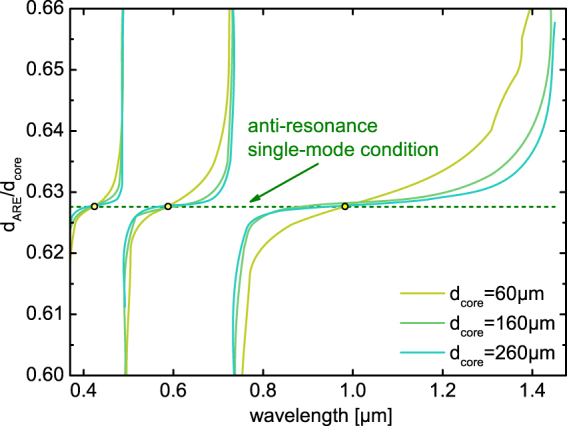
Spectral distribution of the optimal diameter ratio of anti-resonant element and central air core of a revolver-type hollow core fiber to achieve single mode guidance. The ratio was obtained by phase-matching the effective indices of the first higher-order core mode to the fundamental mode of the ARE using Eq. (25). The three different solid lines refer to different central core diameters (yellow: 60 $\mu$m, green 160 $\mu$m, blue: 260 $\mu$m) and the dashed horizontal green line to the criterion derived by Uebel *et al*.^50^. The three yellow dots emphasize the anti-resonant point, i.e., the configurations at which the criterion derived by Uebel *el al*. is valid.

To illustrate the usefulness of our dispersion model, we apply the analytic form of the real part of the effective mode index (Eq. 25) to two different scientific questions addressing important issues of HCF research. The first issue relates to the suppression of HOMs in hollow-core revolver-type fibers (or hypocycloid core contour anti-resonant HCFs) via resonant modal filtering^26,50^. The idea of this approach relies on phase-matching the first higher-order mode in the central air core to the fundamental mode of the surrounding anti-resonant elements (AREs), effectively removing the contributions of the HOMs from the overall fiber transmission and thus yielding single-mode guidance. In the work presented by P. Uebel *et al*.^50^, this phase-matching process was analyzed using the analytic form of the dispersion of a capillary waveguide (air core surrounded by an infinite homogenous glass cladding, Marcatili–Schmeltzer expression^37^), showing that the ratio of the diameters of ARE *d*_*ARE*_ and central core *d*_*core*_ should be approximately 0.628 (in case the two fitting parameters used in the work by Uebel *et al*. are assumed to be unity). It is important to note that this analysis relies on the dispersion equation of a large-core capillary, and thus it remains unclear to what extent resonances influence the condition. Here, we apply the analytic form of the dispersion equation of the tube-type anti-resonant fiber (Eq. 25) within the mentioned phase-matching condition, revealing two key issues: First, the condition derived by Uebel *et al*. only holds exactly at the anti-resonant points (i.e., at those points tube-type fibers behave like capillaries waveguides), and higher or smaller ratios of ${d}_{ARE}/{d}_{core}$ are found towards the edges of the transmission bands (Fig. 7). Second, deviations from the criterion presented by Uebel *et al*. are more pronounced for smaller core diameters, showing that revolver-type fibers with large cores are less critical with regard to single-mode guidance.

A similar kind of consideration can be applied to analyze the critical bend radius of revolver-type HCFs. An analytical equation of the critical bend radius presented in ref.^51^ is based on phase-matching the effective indices of core and capillary mode, whereas the latter is modified by a bend radius dependent factor. The calculations of ref.^51^ include the Marcatili expressions^37^ for Re(${n}_{eff}$) which correspond to our equations at the anti-resonant point (i.e., at $\cot \,\phi =0$).

As mentioned in the previous section, another important application area of HCFs is ultrafast nonlinear light generation in gas-filled HCFs mediated by effects such as soliton fission and the creation of Cherenkov radiation^4^. The most important design aspect for this type of light generation scheme is to engineer and optimize the nonlinear pulse propagation inside the fiber core which strongly depends on the spectral distribution of the GVD of the mode of interest. A key parameter that acts as a design indicator widely used in the supercontinuum generation community and needs to be optimized to achieve the desired properties of the generated light is the wavelength at which the GVD vanishes. In the vicinity of this wavelength, which is commonly referred to ZDW, the propagating pulse is hardly influenced by second-order dispersion, and strong nonlinear spectral broadening can be expected due to the small temporal pulse dispersion.

**Figure 8:**
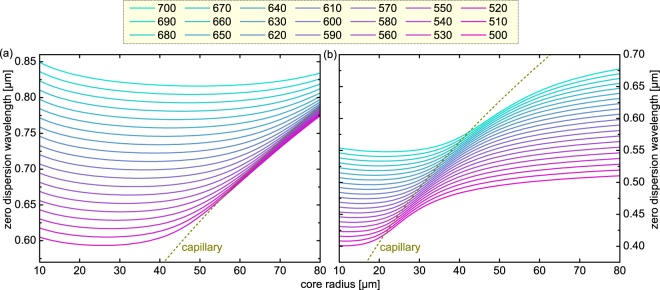
Dependence of the zero-dispersion wavelength (ZDW) on the core radius of the fundamental leaky mode of an argon-filled tube-type anti-resonant hollow core fiber ($T=293$ K, $p=1$ bar) for various values of the tube thickness *w*. The different colors refer to the various thicknesses considered (represented on the top of the plot (in nm)). The two diagrams refer to the behavior of the ZDW in two different transmission bands: (**a**) the transmission band between the resonances *l* = 1 and *l* = 2, (**b**) transmission band between the resonances *l* = 2 and *l* = 3. In both plots, the dark yellow dashed lines refer to the ZDW of an argon-filled capillary of equal core diameter.

The analytic form of Eq. (25) allows studying the dependency of the ZDW of the fundamental leaky mode of the tube-type HCF geometry on structural parameters at different wavelengths as exemplified in the following. Here, we assumed a silica ring located in argon (material dispersions of silica and argon at a temperature and pressure of 273 K and 1 bar are taken from refs^52,53^), and calculate the ZDW by numerically finding the root of the spectral distributions of the GVD in the respective band (i.e., by finding the wavelength at which the GVD changes sign (Fig. 5a)). The results for two bands (located between the *l* = 1 and *l* = 2 resonances (Fig. 8a), and the *l* = 2 and *l* = 3 resonances (Fig. 8b)) clearly illustrate that the presence of the resonances has a dramatic effect on the ZDW compared to the case of an argon-filled capillary (dark yellow dashed lines in Fig. 8). By appropriately choosing the structural parameters (i.e., the core radius and tube thickness), the ZDWs can be tuned to a much greater extent than can be achieved in a capillary. It is important to note that each transmission band includes one ZDW, allowing a single device exhibiting multiple ZDWs to be created, which is in great contrast to a capillary, which typically has only one zero-crossing of the GVD for a given core diameter.

## Conclusion

Fibers with hollow interiors, and in particular anti-resonant HCFs, have recently attracted the attention of the photonics community due to their straightforward fabrication and outstanding guidance performance. Here we introduce a reflection model that approximates the light guidance in tube-type anti-resonant HCFs by the reflection of a wave at a planar film, obtaining entirely analytic expressions for the complex effective index of the supported leaky modes. Comparisons with numerical solutions confirm that the model is valid for any practically relevant combination of core radius, wavelength, and glass RI and enables fundamental insights into the light guidance of tube-type waveguides to be obtained. In particular, excellent agreement was found for the most important dispersion parameters (i.e., phase velocity, GV, and GVD) and for the imaginary parts of the effective indices. Three important facts have been discussed here: (i) the loss of tube-type anti-resonant fibers scale with the inverse fourth power of the core radius ($\propto {R}^{-4}$) at all wavelengths, which is in contrast to other fiber geometries such as Omniguides; (ii) the anti-resonant wavelength and the wavelength of lowest loss do not coincide; and (iii) glass ring RIs below 1.632 are favorable for discriminating the HE_11_ mode. The latter fact is independent of the wavelength, core diameter, and ring thickness and is not visible in the modal dispersions, counterintuitively suggesting the usage of glass materials with low RI to preferentially guide the HE_11_ mode. We apply the analytic form of the phase index to two issues relevant for HCF research. First, we investigate HOM suppression in revolver-type fibers mediated by phase-matching them to modes in the AREs. We found that the single-mode criterion presented in the literature only holds at the anti-resonant point and changes when approaching the transmission band edges, whereas fibers with larger cores show this effect in a less pronounced manner. Second, we show that resonances strongly change the behavior of the ZDW compared to a gas-filled capillary. Due to its universal character and its excellent agreement with numerical solutions our model clearly reveals that the reflection of a single wave on a planar film is the essential key to understanding the properties of leaky modes supported by tube-type waveguides. It is important to note that first transmission experiments on thin-wall capillaries at visible wavelengths clearly indicate the potential of hollow tube-type anti-resonant waveguides with applications in multiple fields^43^. The revealed inverse fourth power core radius dependence shows that the loss of anti-resonant fibers fundamentally scales differently compared to other geometries such as photonic band gap fibers or capillaries, whereas precise studies are required to specifically explain the behavior of one particular anti-resonant fiber geometry. The two discussed applications of the analytic expression of the real part of the effective mode index of the supported leaky mode to scientific questions being highly relevant for HCF research represent only two selected examples. At this stage, we believe that our analytic model can also be applied to other types of problems related to light guidance in thin-wall hollow cylinders. Extending our model to other types of hollow waveguides including photonic band gap fibers, Bragg fibers or Omniguides, or even planar geometries will be the subject of future studies and will allow further insights to be obtained into light guidance in hollow waveguides and the properties of leaky modes in general. Moreover, we believe that a modified version of our approach will be useful for optimizing the performance of anti-resonant fibers by calculating the reflection coefficient of the cladding numerically and using the derived equations to calculate the loss, eliminating the need to solve the eigenmodes of the entire fiber cross section.

## Methods

### Numerical solutions for comparison

Full numerical solutions were obtained using the solution of the Helmholtz equation in cylindrical coordinates^46^. To account for the leakiness of the modes in anti-resonant HCFs, we used a Hankel function of the first kind in the most outer medium in order to represent outgoing waves. With this ansatz the boundary conditions at both interfaces result in a system of four linear equations in the case of TE or TM modes and eight equations for the hybrid modes. The values of the complex effective index that represent the modes are obtained by numerically finding the roots of the determinants of the system of equations.

### Expressions of the field

The general solutions of the Helmholtz equation in cylindrical coordinates ($\rho$, $\phi$, *z*) can be expressed as

28 $${\boldsymbol{E}}(\rho ,\phi ,z)=[{E}_{\rho }(\rho ){{\boldsymbol{e}}}_{\rho }+{E}_{\phi }(\rho ){{\boldsymbol{e}}}_{\phi }+{E}_{z}(\rho ){{\boldsymbol{e}}}_{z}]\,\exp [i(\beta z+m\phi -\omega t)],$$

29 $${\boldsymbol{H}}(\rho ,\phi ,z)=[{H}_{\rho }(\rho ){{\boldsymbol{e}}}_{\rho }+{H}_{\phi }(\rho ){{\boldsymbol{e}}}_{\phi }+{H}_{z}(\rho ){{\boldsymbol{e}}}_{z}]\,\exp [i(\beta z+m\phi -\omega t)],$$

where ${{\boldsymbol{e}}}_{\rho }$, ${{\boldsymbol{e}}}_{\phi }$, and ${{\boldsymbol{e}}}_{z}$ are the unity vectors in cylindrical coordinates. The full expressions of the radial functions ${E}_{\rho }(\rho )$, ${E}_{\phi }(\rho )$, ${E}_{z}(\rho )$, ${H}_{\rho }(\rho )$, ${H}_{\phi }(\rho )$ and ${H}_{z}(\rho )$, containing Bessel functions of the first (*J*_*m*_) and second (*Y*_*m*_) kind, are given in ref.^46^. To obtain a physically finite solution, we drop all the terms including *Y*_*m*_ because this functions exhibits a singularity at the origin ($\rho =0$). The exponential factors in Eqs (28) and (29) are identical in all media and thus can be neglected. Because only the tangential field components are required to fulfill the boundary conditions, the only relevant factors to be considered for the mathematical analysis are those given in Eqs (1–2).

### Relations of the Bessel functions

The perturbation treatment conducted above requires calculation of the first, second, and third derivatives of the Bessel function *J*_*m*_ for arguments that are zeros of the Bessel function ${J}_{m\pm 1}$, which are analytically expressed as follows. The first derivative of *J*_*m*_ is given by^54^

30 $${J}_{m}^{^{\prime} }(x)=\frac{1}{2}({J}_{m-1}(x)-{J}_{m+1}(x))\mathrm{.}$$

Applying Eq. (30) recursively allows higher derivatives to be transformed into analytic expressions including multiple Bessel functions of different order, which can be further reduced by applying the following equation^54^ repeatedly:

31 $${J}_{m-1}(x)+{J}_{m+1}(x)=\frac{2m}{x}{J}_{m}(x\mathrm{).}$$

With this procedure, any derivative of the *J*_*m*_ function can be transformed into the following type of expression:

32 $${J}_{m}^{(k)}(x)=p(x){J}_{m}(x)+q(x){J}_{m+s}(x),$$

where *p*(*x*) and *q*(*x*) are rational functions and $s=\pm 1$. In particular, the first three derivatives of the *J*_*m*_ function can be expressed as follows:

33 $${J}_{m}^{^{\prime} }(x)=\frac{sm}{x}{J}_{m}(x)-s{J}_{m+s}(x),$$

34 $${J}_{m}^{^{\prime\prime} }(x)=\frac{{m}^{2}-sm-{x}^{2}}{{x}^{2}}{J}_{m}(x)+\frac{s}{x}{J}_{m+s}(x),$$

35 $${J\prime\prime\prime }_{m}(x)=\frac{(sm-\mathrm{1)}({m}^{2}-2sm-{x}^{2})}{{x}^{3}}{J}_{m}(x)+\frac{s}{{x}^{2}}({x}^{2}-2-{m}^{2}){J}_{m+s}(x\mathrm{).}$$

If the argument is the *n*-th zero of *J*_*m*+s_ we obtain

36 $${J}_{m}^{^{\prime} }({j}_{m+s,n})=\frac{sm}{{j}_{m+s,n}}{J}_{m}({j}_{m+s,n}),$$

37 $${J}_{m}^{^{\prime\prime} }({j}_{m+s,n})=\frac{{m}^{2}-sm-{j}_{m+s,n}^{2}}{{j}_{m+s,n}^{2}}{J}_{m}({j}_{m+s,n}),$$

38 $${J\prime\prime\prime }_{m}({j}_{m+s,n})=\frac{(sm-\mathrm{1)(}{m}^{2}-2sm-{j}_{m+s,n}^{2})}{{j}_{m+s,n}^{3}}\cdot {J}_{m}({j}_{m+s,n}\mathrm{).}$$

### Data availability

All data generated or analyzed during this study are included in the figures and can be reproduced using the equations of this published article.
