# Author Correction: Analytic model for the complex effective index of the leaky modes of tube-type anti-resonant hollow core fibers

**DOI:** 10.1038/s41598-018-19722-2

**Published:** 2018-01-24

**Authors:** Matthias Zeisberger, Markus A. Schmidt

**Affiliations:** 1 Leibniz Institute of Photonic Technology, Albert-Einstein-Str. 9, 07745 Jena, Germany; 2 Otto Schott Institute of Materials Research (OSIM), Friedrich Schiller University of Jena, Fraunhoferstr. 6, 07743 Jena, Germany; 3 Abbe Center of Photonics and Faculty of Physics, Friedrich Schiller University Jena, Max-Wien-Platz 1, Jena, 07743 Germany

Correction to: *Scientific Reports* 10.1038/s41598-017-12234-5, published online 18 September 2017

This Article contains errors within Figure 7, in which certain curves are distorted. The correct Figure 7 appears below as Figure 1:

**Figure 1:**
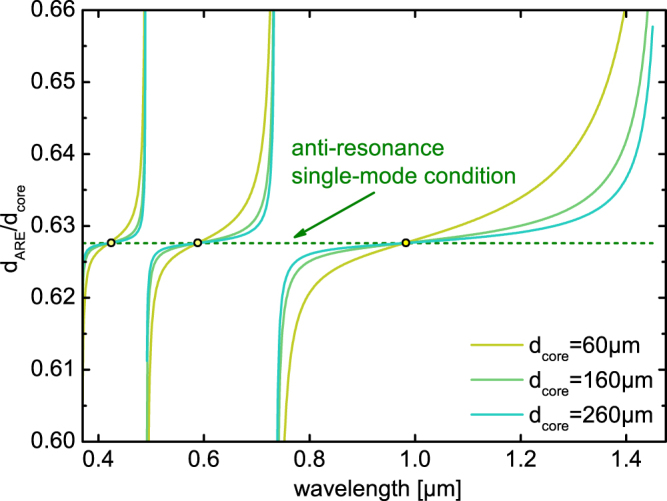
.

